# A real-world workplace-based screening for *Helicobacter pylori* infection: the HPOS study

**DOI:** 10.1007/s10552-026-02189-2

**Published:** 2026-07-30

**Authors:** Giulia Collatuzzo, Giulia Fiorini, Matteo Pavoni, Maria Vittoria Costanzucci Paolino, Andrei Cosmin Siea, Maria Zafeiratou, Laura Astolfi, Luca Magni, Tommaso Renieri, Silvia Mangiaterra, Marika D’Agostini, Nataliia Danilevskaia, Dino Vaira, Paolo Boffetta

**Affiliations:** 1 Department of Biomedical and Clinical Sciences (DIBIC), University of Milan, Milan, Italy; 2 IRCCS Azienda Ospedaliero-Universitaria di Bologna , Bologna, Italy; 3 Department of Medical and Surgical Sciences, University of Bologna, Bologna, Italy; 4 Department of Family, Population and Preventive Medicine, Renaissance School of Medicine, Stony Brook University, Stony Brook, NY USA; 5 Stony Brook Cancer Center, Stony Brook University, Stony Brook, NY USA

**Keywords:** *Helicobacter pylori*, Screening, Gastric cancer prevention, Stool antigen test, Workplace, Occupational health surveillance, Total worker health

## Abstract

**Objectives:**

*Helicobacter pylori* (*H.pylori*) eradication contributes to gastric cancer (GC) prevention, yet many subjects are unaware of their infection status. We implemented a workplace-based *H.pylori* screening.

**Design:**

We implemented a cross-sectional screening with short-term follow-up among hospital workers (HWs), enrolled during occupational health surveillance (OHS). A stool antigen test (SAT) was adopted for *H.pylori* diagnosis. *H.pylori*-positive HWs were referred to their general practitioner (GP) or a gastroenterologist and were followed up at three months. Participation, prevalence of infection, participants’ experience, and treatment rates were investigated through descriptive and multivariable analyses.

**Setting:**

The study was carried out between 2023 and 2025 at S.Orsola University Hospital in Bologna, Italy.

**Participants:**

The study targeted HWs aged 40–65 employed at S.Orsola University Hospital.

**Results:**

Of 744 eligible HWs contacted, 550 were enrolled (73.9%), including 399 through OHS and 151 volunteers; the largest proportion of participants were women (74.7%) and nurses (40.2%). Dropout rate was 15.5% (*n* = 115), leaving 435 HWs with a SAT result. *H.pylori* prevalence was 16.6%. Men were less likely to participate than women (OR = 0.68, 95% CI = 0.47–0.99). Education was negatively associated with *H. pylori* infection (OR = 0.41, 95% CI = 0.18–0.93 for the highest educational level vs the lowest). No specific pattern was observed in relation to contact with a physician and treatment prescription. 58/72 *H. pylori*-positive HWs (80.6%) contacted a physician, 53 (73.6%) being prescribed a treatment and 13 (18.1%) undergoing upper endoscopy. Confirmatory test rate after treatment was suboptimal, while eradication rate was 97.3% when considering those with confirmatory test result (36/37 HWs). Across followed up HWs, the average quality of experience was 8.9/10; 13 had a family member tested following this intervention.

**Conclusions:**

The intervention integrated into OHS was well received and extended to family members of the participants, suggesting that a workplace-based *H.pylori* screening might contribute to GC prevention. Replications in other workplaces are warranted.

**Supplementary Information:**

The online version contains supplementary material available at https://doi.org/10.1007/s10552-026-02189-2.

## Introduction

Occupational medicine (OM) is dedicated to prevention, diagnosis, and management of work-related diseases and injuries [1]. Its aims include assessing occupational risks and promoting a safe and healthy work environment [1]. According to the concept of Total Worker Health (TWH) [2], the potential of OM in contributing to public health extends beyond work-related diseases. Workplace-based cancer screening is part of a TWH approach. In fact, the workplace might represent an ideal setting for the implementation of cancer screenings given the structured, periodical character of occupational health surveillance (OHS), which is mandatory and free for all workers [3, 4].

*Helicobacter pylori* (*H.pylori*) infection is the leading cause of infection-related cancer worldwide, being the primary risk factor for gastric cancer (GC) [5]. In Italy, *H.pylori* was estimated to have caused 2.4% of total cancer cases and 3.3% of cancer deaths in 2020 [6]. The non-invasiveness of *H.pylori* detection methods and the availability of antibiotic-based therapies make GC preventable through *H.pylori* test-and-treat strategies [7]. Despite clear evidence supporting the effectiveness of these strategies for GC prevention, no standardized large-scale *H.pylori* screening has been implemented yet [8], also resulting in low awareness of *H.pylori*-related risks [9–12].

Recent literature highlights the need for GC prevention in Europe [13]. A targeted approach might represent a facilitator for the successful implementation of an *H.pylori* screening. In particular, workplace-based *H.pylori* screening has been previously proposed [4, 14].

The HPOS (*H.pylori* screening among hospital workers [HWs]) study was established to assess the feasibility and acceptability of a workplace-based *H.pylori* screening as part of the OHS. In addition, the study aimed at describing the prevalence of *H.pylori* infection in a population of Italian HW, and evaluating the impact of a workplace-based *H.pylori* screening at 3 months from diagnosis.

HPOS is the pilot study of the cancer prevention at work (CPW), a multicenter international project currently ongoing in Europe, which includes the implementation of *H.pylori* screening across different workplace settings [15].

In this paper, we illustrate the design, results, and impact of the HPOS study.

## Methods

The HPOS study is a cross-sectional interventional study based in Bologna, Italy, targeting HWs. The study was developed by a multidisciplinary team of occupational health providers and gastroenterologists from the University of Bologna and the IRCSS S.Orsola University Hospital. The study was approved by the local Ethics Committee and was conducted between March 2023 and March 2025. The estimated target was 496 workers, based on an expected *H.pylori* prevalence of 25.0% and a 10.0% dropout rate (defined as missing SAT return for analysis).

The OM Unit in Italy operates in compliance with regulations [16] mandating that all employees undergo baseline medical examination to assess their fitness for work. In brief, the intervention consisted of *H.pylori* screening via stool antigen test (SAT) offered during routine OHS, supported by educational materials to raise awareness on *H.pylori*-related diseases, including GC. *H.pylori*-positive HWs received a written report, including recommendation on *H.pylori* management, and were referred to their general practitioner (GP); they were followed up at three months to investigate infection management, collect feedback, and to support family members interested in *H.pylori* screening.

Before the study commenced, informational sessions were organized for all OM providers to outline the study process. Additionally, training sessions were conducted for the research team to ensure protocol adherence throughout all phases. Enrollment progress was regularly reviewed, and issues were promptly addressed.

### Study population

The target population consisted of HWs employed at S. Orsola University Hospital, Bologna, Italy. Next to employment at that hospital, inclusion criteria were age 40–65 and provision of a written consent form. Age range was established based on (i) birth cohort effect of *H.pylori* prevalence, with increasing likelihood of infection with age [17]; (ii) possible expanded effect on workers’ adult family members [18]; (iii) rationale for eradication treatment. Exclusion criteria were pregnancy/breastfeeding, gastrectomy, active cancer and severe disease condition, and rectal bleeding/diarrhea. Workers who were short-term users of proton pump inhibitors (PPI) or antibiotics were temporarily excluded and considered for enrollment after four weeks from the end of therapy [8]; similarly, those using antacid were considered for enrollment after 10 days from stopping the treatment [8]. Workers willing to participate despite chronic therapy with PPI were not recommended to interrupt their therapy and were referred to their GP. The remaining subjects under chronic PPI treatment were considered ineligible.

Workers who had ever been tested or treated for *H.pylori* infection were not excluded, given that our aim was to describe the prevalence of infection within this population.

### Enrollment strategies

**Fig. 1:**
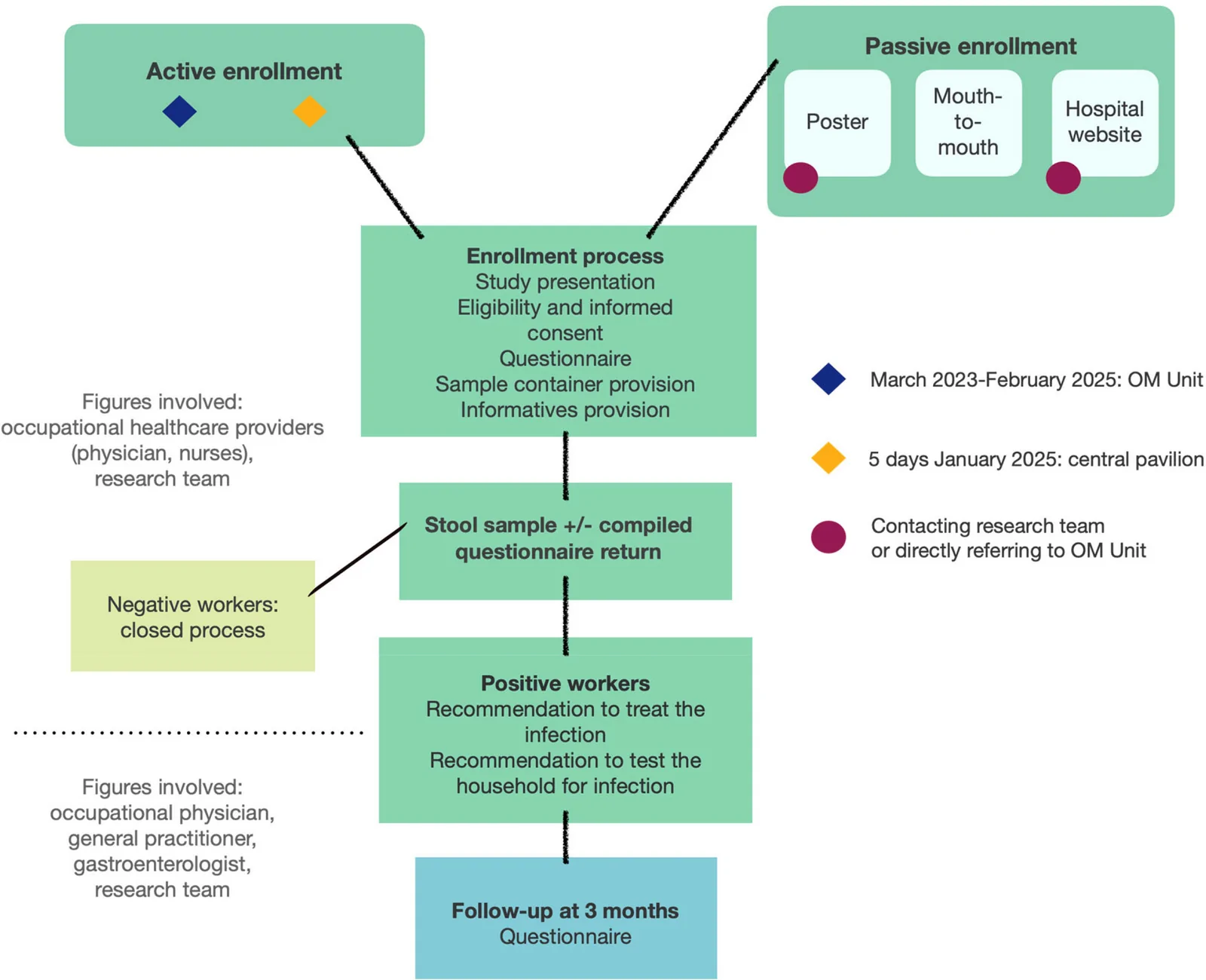
Scheme of the HPOS intervention

Figure 1 illustrates the enrollment strategies adopted within the HPOS intervention scheme. The study was supported by communication materials: posters presenting the study were placed at the entrance of the OM Unit, at the reception desk and in strategic areas such as the canteen and the main hospital pavilion. Two types of enrollment could be distinguished: (i) workers recruited at the moment of the regular OHS, either in the waiting room or during the visit (active enrollment); (ii) workers who spontaneously referred to the research team following either information posted on posters and online, or recommendations from colleagues (volunteer enrollment). The analyses were conducted considering the combination of all enrolled participants, namely active enrollment as well as volunteers. The latter group was also described by key characteristics.

Participants were invited to the study in a neutral context, without coercion or perception of a possible influence of participation on their working conditions. In fact, many workers wanted to take part in the study as volunteers following poster visualization or mouth-to-mouth reporting, as the implemented intervention was purposed as a free opportunity integrated in OHS.

Documents provided to the HWs are included in Appendices A–E. A trained researcher illustrated the study to potential participants, also providing informative materials (Appendices A and B) if they were interested. Workers who agreed to participate signed an informed consent (Appendix C), were given a numerical code, and completed a questionnaire (details below). A stool sample kit was provided, together with specific information on its use, storage, and return. The study design specifically instructed participants to return their stool samples to the OM department within 48 h of collection, to ensure that the samples were analyzed within 24 h of receipt.

### Questionnaires, communication of test result, and three-month follow-up

The baseline questionnaire (Appendix D) comprised 8 sections: sociodemographic, occupational, lifestyle, dietary, *H.pylori* infection knowledge and personal *H.pylori* history, gastric disease history, other clinical history, and gastrointestinal symptoms. The follow-up questionnaire (Appendix E) investigated whether the HWs had made contact with a physician to manage *H.pylori* infection, treatment undergone and further testing (including the type of test to confirm successful eradication), change in health status, and feedback on the study experience. Participants filled out the paper questionnaire at the time of enrollment. They were given the option to fill out the questionnaire at home and return it with the stool sample. Data of the compiled questionnaires were then de-identified and inserted into the dedicated database.

SAT returned by the HWs were stored in a fridge at the OM Unit before transfer to the laboratory, which was located close to the OM Department. In particular, stool specimens were stored at 4 °C for no longer than 48 h. The detection of *H.pylori* antigen was carried out using the Curian® HpSA® assay (Meridian Bioscience Europe, Milan, Italy), a rapid, qualitative fluorescent immunoassay specifically designed for human stool samples, with an analytical turnaround time of approximately 20 min [19–21]. At the beginning of each analytical session, one positive control and one negative control were included to verify assay reliability. Test results were recorded in the dedicated database.

To increase compliance for returning the SAT and questionnaire (if not completed at enrollment), each participant was solicited by phone call up to 3 times. HWs who agreed to participate in the study but did not return either the questionnaire or the SAT were considered dropouts. 

SAT results were communicated via e-mail, with the official report attached. In the event of a positive result, the report was preceded by a phone call during which the worker was informed of the diagnosis and advised to consult the GP for continuation of the therapeutic pathway. Privacy of test results was maintained with communication of the result from the researcher solely to the worker, via the phone number and e-mail contact provided at the time of enrollment. Employers, colleagues, and occupational physicians not involved in the present study did not receive the SAT results of the participants, unless the workers themselves expressed the intention to disclose them. Similarly, the SAT result was not communicated to third parties, unless the worker asked to put the research team directly in contact with family members or the GP. The SAT result was not used and was not mentioned in the report for the fitness to work.

After three months, positive HWs were recontacted by phone for the follow-up interview. Notably, while evidence-based recommendations were provided to workers screened positive to SAT, the following *H.pylori* management varied based on the GP’s or the gastroenterologist’s rationale. Therefore, the information collected at the follow-up is self-reported data, not necessarily supported by medical documentation, not reflecting standardized procedure, but rather a real-world, heterogeneous pathway of *H.pylori* management. This allowed us to capture the adherence of actual *H.pylori* management to current guidelines.

Operational details for replication of the intervention are available in Appendix F.

### Statistical analysis

We distinguished three age groups, namely 40–45, 46–55, and 56–65. Job titles were coded as nurses, health assistants, physicians/researchers (including staff physycians, researchers, resident physicians and PhD students), technicians and other health professionals, and non-healthcare workers (including office workers, maintenance workers, cooks, and gardeners).

Gastroenterology and endoscopy departments were considered at high risk for *H.pylori* infection [22]. HWs were considered symptomatic when they referred to at least one of the following disturbances: nausea, epigastric discomfort/pain, regurgitation, or heartburn.

Descriptive analyses were performed by engagement status and by *H.pylori* status. Chi-square tests among categories were computed. Multivariate logistic regression models were run to investigate the determinants of enrollment (adjustments: sex, age, and job title), as well as determinants of contact with a physician (adjustments: sex, age, job title, and symptoms) and treatment prescription among the *H.pylori*-positive HWs (adjustments: sex, age, and symptoms) expressed as odds ratio (OR) and 95% confidence interval (CI). We also analyzed prevalence and determinants of *H.pylori* infection through models adjusted by sex and age.

Descriptive analyses were conducted to explore the impact of *H.pylori* diagnosis and our intervention after three months (including contact with a physician, treatment prescription, test to confirm the successful eradication, documented eradication rate, *H. pylori* screening extended to family members, and overall feedback).

Statistical analyses were conducted using Stata v16 [23].

The STROBE checklist is provided in Supplementary Table 1.

## Results

**Fig. 2:**
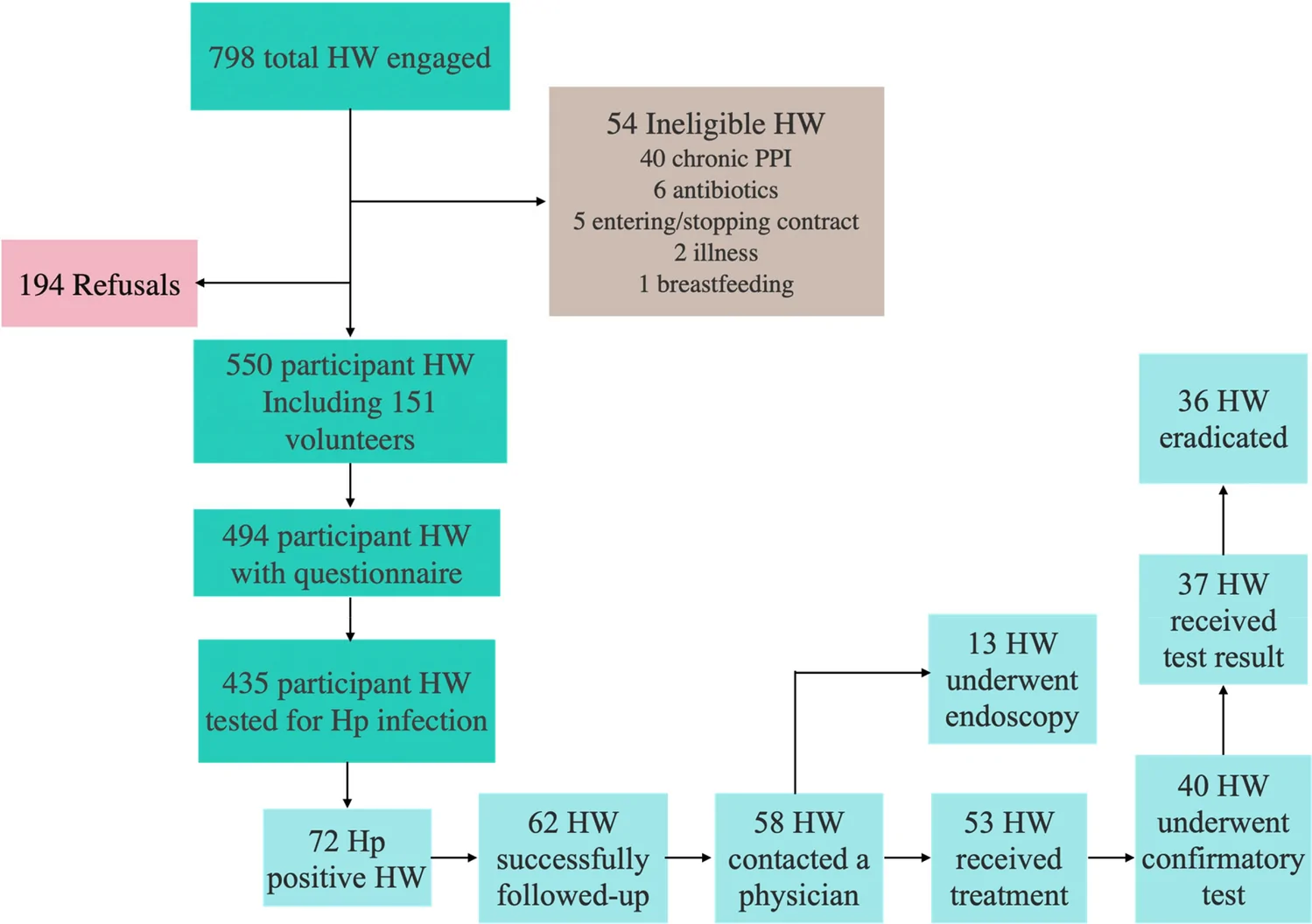
Flow chart of the recruitment of HPOS study participants

Of a total of 744 eligible HWs, 550 were enrolled (73.9%), in particular 399 during OHS and additional 151 as volunteers, and 194 declined participation (26.1%). Overall, 494 HWs filled the questionnaire and 435 were successfully tested for *H.pylori* through SAT, while 115 HWs dropped from the study. Figure 2 illustrates the flow diagram of the recruitment process.

Table 1 shows the characteristics of the study population. HWs contacted were in large part women (*n* = 534, 71.8%), aged 46–55 (*n* = 311, 41.8%), employed as nurses (*n* = 299, 19.9%). Enrolled HWs were mainly women (411, 74.7%, *p*-het = 0.003), aged 46–55 (235, 42.7%, *p*-het = 0.573), and nurses (*n* = 221, 40.2%, *p*-het = 0.704).

**Table 1:** Characteristics of the study population overall and by participation status

Population	Sex *n*, (%)		Age *n*, (%)			Job title *n*, (%)				
	Women	Men	40–45	46–55	56–65	Nurse	Health assistant	Physician/ researcher	Technicians and other hcp	Non-hcp
Contacted (*n* = 744, 100%)	534 (71.8)	210 (28.2)	201 (27.0)	311 (41.8)	232 (31.2)	299 (40.2)	148 (19.9)	124 (16.6)	127 (17.1)	46 (6.2)
*p-value*	< *0.001*		< *0.001*			< *0.001*				
Enrolled (*n* = 550, 73.9)	411 (74.7) (77.0)	139 ( 25.3) (66.2)	149 (27.1) (74.1)	235 (42.7) (75.6)	166 (30.2) (71.5)	221 (40.2) (73.9)	116 (21.1) (78.4)	79 (14.4) (63.7)	101 (18.4) (79.5)	33 (6.0) (71.4)
*p-value*	*0.003*		*0.573*			*0.704*				
Enrolled through OHS (*n* = 399, 53.6%)	291 (72.9%) (54.5%)*	108 (27.1%) (51.4%)*	107 (26.8%) (53.2%)*	172 (43.1%) (55.3%)*	120 (30.1%)(51.7%)*	148 (37.1%) (49.5%)*	84 (21.0%) (56.8%)*	66 (16.5%) (53.2%)*	78 (19.6%) (61.4%)*	23 (5.8%) (50.0%)*
*p-value*	*0.02*		*0.57*			*0.10*				
Refusals (*n* = 194, 26.1%)	123 (63.4%) (23.0%)*	71 (36.6%) (33.8%)*	52 (26.8%) (25.9%)*	76 (39.2%) (24.4%)*	66 (34.0%) (28.4%)*	78 (40.2%) (26.1%)*	32 (16.5%) (21.6%)*	45 (23.2%) (36.3%)*	26 (13.4%) (20.5%)*	13 (6.7%) (28.3%)*
*p-value*	*0.02*		*0.57*			*0.08*				
Volunteers (*n* = 151, 20.3%)	120 (79.5%) (22.5)	31 (20.5%) (14.8)	42 (27.8%) (20.9)	63 (41.7%) (20.3)	46 (30.5%) (19.8)	73 (48.3%) (24.4)	32 (21.2%) (21.6)	13 (8.6%) (10.4)	23 (15.2%) (18.1)	10 (6.6%) (21.7)
*p*-*value*	0.02		1.00			0.03				
Dropouts ϕ (N = 115, 15.5%)	76 (66.1%) (14.2%)*	39 (33.9%) (18.6%)*	41 (35.6%) (20.4%)*	40 (34.8%) (12.9%)*	34 (29.6%) (14.7%)*	35 (30.4%) (11.7%)*	30 (26.9%) (20.3%)*	32 (27.8%) (25.8%)*	14 (12.7%) (11.0%)*	4 (3.5%) (8.7%)*
*P-value*	*0.02*		*0.05*			< *0.001*				

*hcp* healthcare professionals

^*^First percentage refers to row, second percentage to subjects with the characteristics under analysis over total

ϕOf the 115 total dropouts, 10 were recorded among volunteer HW and 105 among HW recruited at OHS

In particular, a higher proportion of women accepted to be enrolled compared with men (77.0% vs 66.2%, *p* =0.003). We observed the highest enrollment rate among technicians and other healthcare professionals (79.5%) and health assistants (78.4%), while the lowest was among physicians/researchers (63.7%). Among the non-participants, females accounted for the majority (63.4%), HWs aged 46–55 comprised 39.2%, and nursing staff accounted for 40.2%. The proportion of refusals was higher in men (33.8%) than in women (23.0%). The refusal rate followed the order physician/researcher > non-healthcare professionals > nurses > health assistants > technicians and other healthcare professionals.

When focusing on the 151 volunteers, 120 were women (79.5%). Again, most were aged 46–55 (41.7%) and worked as nurses (48.3%) or health assistants (21.2%). When considering dropouts, men were slightly more likely to drop than women (18.6% vs 14.2%, *p*=0.02), as well as younger HWs subgroup was compared to the others (20.4% of those 40–45 vs 14.7% among 56–55 and 12.9% among 46–55 year-old, *p*=0.05). The largest proportion of HWs who dropped the study was observed among physicians/researchers (25.8%), followed by health assistants (20.3%).

Of the 115 dropouts, 10 were recorded among volunteers (8.7%) and 105 among actively recruited HWs (91.3%).

The results of the multivariate analysis of determinants of enrollment are reported in Table 2: men were significantly less likely to engage in the study compared with women (OR = 0.68, 95% CI = 0.47–0.99). No additional pattern was observed by age and job title.

**Table 2:** Determinants of screening participation and contact with physician and treatment among *Helicobacter pylori* positives—the HPOS study

Characteristic	Outcome
**N = 744**	Enrollment* **OR, 95% CI**
Sex	Ref
- Women	0.68, 0.47–0.99
- Men	
Age	Ref
- 40–45	1.04, 0.67–1.61
- 46–55	0.86, 0.54–1.35
- 56–65	
Job title	
- Nurse	Ref
- Health assistant	1.37, 0.83–2.24
- Physicians/researcher	0.83, 0.51–1.34
- Technicians and other healthcare professional	1.62, 0.96–2.73
- Non-healthcare professional	0.99, 0.47–2.06
**N =** **72**	**Contact with a physician**
Sex	
- Women	Ref
- Men	1.88, 0.43–8.29
Age	
- 40–45	Ref
- 46–55	0.80, 0.20–3.18
- 56–65	0.16, 0.02–1.06
Job title - Nurse - Health assistant - Physicians/researcher - Technicians and other healthcare professional - Non-healthcare professional	
	Ref
	0.68, 0.15–3.09
	3.73, 0.49–28.3
	0.45, 0.07–2.78
	8.25, 0.61–111.3
Symptomatic - No - Yes	
	Ref
	0.70, 0.21–2.32
**N = 72 **	**Treatment prescription**
Sex - Women - Men	
	Ref
	1.79, 0.35–9.24
Age - 40–45 - 46–55 - 56–65	
	Ref
	1.64, 0.41–6.54
	0.95, 0.25–3.65
Symptomatic - No - Yes	
	Ref
	1.04, 0.35–3.02

Note: *N* number, *OR* odds ratio, *CI* confidence intervals, *Ref* reference category;

Multivariate model adjusted for the variables listed for each outcome.

The analysis on knowledge, history, and determinants of *H.pylori* infection was conducted on 494 HWs with available questionnaire data (Supplementary Table 2). Among them, 32 (6.5%) reported to be unaware of *H.pylori* infection as a health issue at the time of the study. When asked “What is the level of knowledge of the *H.pylori* infection among the general population?”, the most frequent answer was “poorly known” (38.1%), and the second was “well known” (34.2%). Most HWs reported to have learned about *H.pylori* during the studies/at the workplace (72.5%), rather than within their family (8.3%), from the GP (6.7%) or from the media (7.0%). A total of 17.2% reported a first-degree family member who was *H.pylori*-positive, and 11.3% reported a family history of GC.

Table 3 presents the relationship between *H.pylori* infection and sociodemographic, lifestyle, and occupational characteristics. Of 435 screened individuals, 72 (16.6%) resulted *H.pylori*-positive, including 49 HWs enrolled during OHS and 23 volunteers. No statistically significant sex or age differences were observed, despite a slightly higher prevalence of infection in women than in men and in the oldest group. Prevalence of *H.pylori* did not differ between HWs enrolled as volunteers and those enrolled at OHS. Health assistants showed the highest *H.pylori* prevalence (24.4%) and physician/researchers showed the lowest (10.6%), although these differences were not statistically significant. *H.pylori* prevalence was inversely associated with educational level (OR = 0.41, 95% CI = 0.18–0.93 for the highest educational level vs the lowest). A borderline higher risk of *H.pylori* infection was observed for separated/widowed workers compared to married/in couple workers. When considering occupational factors, those reporting main laboratory activity had a lower risk of *H.pylori* infection compared to HWs working in clinical or surgical departments (OR = 0.38, 95% CI = 0.14–1.03). Lifestyle habits were not associated with *H.pylori* prevalence in the study population, nor were upper-GI symptoms.

**Table 3:** Odds ratios and 95% confidence intervals of *Helicobacter pylori* infection for selected characteristics

Characteristic* (N = 435)	Workers screened for H.pylori with SAT	
	H.pylori % (N)	OR (95% CI)
Overall	16.6 (72)	
Sex Women Men		
		Ref
	18.2 (61)	
	11.0 (11)	0.57 (0.28–1.13)
Age 40–45 46–55 56–65		
	15.7 (17)	Ref
	14.9 (29)	0.91 (0.48–1.76)
	19.7 (26)	1.25 (0.64–2.46)
Volunteer No Yes		
	16.7 (49)	Ref
	16.3 (23)	0.96 (0.56–1.65)
Job title Nurse Health assistant Physician/researcher Technicians and other hcp Non-hcp		
	16.7 (31)	Ref
	24.4 (21)	1.59 (0.85–2.98)
	10.6 (5)	0.64 (0.23–1.77)
	12.6 (11)	0.72 (0.34–1.52)
	13.8 (4)	0.78 (0.25–2.44)
Education Elementary/middle/technical high school High school University degree Master/PhD		
	22.3 (35)	Ref
	19.0 (4)	0.94 (0.29–3.03)
	15.9 (22)	0.75 (0.40–1.42)
	9.2 (9)	0.41 (0.18–0.93)
Marital status Married/couple Single Separated/widowed		
	14.9 (43)	Ref
	16.0 (12)	1.16 (0.57–2.34)
	25.9 (15)	1.88 (0.94–3.75)
Siblings No Yes		
	17.0 (22)	Ref
	16.0 (47)	0.84 (0.48–1.49)
Family history of *H.pylori* No I do not know Yes		
	19.0 (44)	Ref
	13.0 (14)	0.61 (0.31–1.17)
	15.8 (12)	0.75 (0.37–1.53)
Smoking Never Former Current		
	11.6 (11)	Ref
	18.5 (42)	1.62 (0.79–3.32)
	16.5 (17)	1.42 (0.62–3.22)
BMI -< 25 25–29.9 ≥ *30*		
	14.5 (34)	Ref
	18.1 (21)	1.32 (0.72–2.44)
	20.0 (17)	1.42 (0.74–2.71)
Main working activity Clinical department/surgery Clinical consultancy Laboratory Office/other		
	20.1 (35)	Ref
	16.7 (21)	0.70 (0.38–1.28)
	9.4 (5)	0.38 (0.14–1.03)
	16.1 (9)	0.70 (0.30–1.59)
High-risk department No Yes		
	16.6 (59)	Ref
	15.6 (10)	0.93 (0.44–1.94)
Invasive procedures No Yes		
	15.6 (39)	Ref
	18.1 (31)	1.27 (0.75–2.16)
Alcohol No 1–4 unit/week 5 + unit/week		
	17.9 (24)	Ref
	19.6 (27)	1.28 (0.68–2.40)
	11.3 (6)	0.67 (0.25–1.78)
Physical activity None Low Moderate Intense		
	16.1 (14)	Ref
	14.0 (16)	0.89 (0.41–1.95)
	19.1 (37)	1.28 (0.64–2.54)
	12.5 (3)	0.91 (0.23–3.52)
Abdominal pain in the last 3 months No Yes		
	17.5 (42)	Ref
	16.4 (28)	0.88 (0.52–1.50)

Notes: N, number; OR, odds ratio; CI, confidence intervals, Ref, reference category; BMI, body mass index; hcp, healthcare professionals

Multivariate model adjusted for sex and age

^*^High-risk departments: digestive or respiratory endoscopy department

Table 4 summarizes the data collected at three-month follow-up. Of the 72 *H.pylori*-positive HWs, 10 (13.9%) did not answer the phone calls or dropped from the study, leaving 62 HWs who were followed up (86.1%). Four of the 72 *H.pylori*-positive HWs did not contact any doctor (5.6%) and 58 did (80.6%); in particular, the majority contacted their GP (67.2%) and the remaining contacted a gastroenterologist (32.8%). Of those who contacted a physician, 13 HWs underwent upper endoscopy (22.4%), 11 being diagnosed with atrophic gastritis, one with erosive gastritis, and one found negative at further *H.pylori* testing but with gastric polyposis. No particular association with symptoms or age in those referred to endoscopy was observed.

**Table 4:** Follow-up of *Helicobacter pylori*-positive hospital workers

Population of H.pylori-positive HW	N (%)	Comments
H.pylori-positive HW who were successfully contacted for the diagnosis response	72 (100)	One H.pylori-positive HW was excluded before follow-up because we were never able to contact this subject to discuss the positive test result.
Sex - Women - Men		
	61/72 (84.7)	
	11/72 (15.3)	
Mean age	51.8, 50.2–53.5	
Job title - Nurse - Health assistant - Physicians/researcher - Technicians and other healthcare professional - Non-healthcare professional		
	31/72 (43.1)	Other health professionals included physiotherapists, obstetrics, psychologists, biologists, medical physics, dietitian, pharmacists, veterinary, speech-therapist. Non-healthcare professionals included kitchen workers, gardeners, administrative staff.
	21/72 (29.2)	
	5/72 (6.9)	
	11/72 (15.3)	
	4/72 (5.6)	
Volunteers	23/72 (31.9)	
Dropout - No - Yes		Eight HW never answered the phone while two HW asked to be dropped.
	62/72 (86.1)	
	10/72 (13.9)	
Symptomatic - No - Yes		HW considered symptomatic when reported nausea, epigastric discomfort/ pain, regurgitation, or heartburn.
	27/72 (37.5)	
	42/72 (58.3)	
Contact with physician		
- Yes	4/72 (5.6)	In particular, 39 HW contacted the GP (67.2%), 19 contacted the gastroenterologist (32.8%).
- No	58/72 (80.6)	One HW explained not to be willing to undergo eradication treatment because she already did it in the past, and not to have contacted her GP.
- Missing	10 (13.9)	One HW, asymptomatic, explained to have procrastinated the contact with any physician despite willing to.
Upper endoscopy		
- No	45/72 (62.5)	Histological reports: 11 atrophic gastritis; one erosive gastritis; one H.pylori negative with gastric polyposis. Upper endoscopy performed in 5 asymptomatic and 8 symptomatic HW. Of the asymptomatic, 2 were aged 40–45, 2 were 46–55, 1 was 56 +.
- Yes	13/72 (18.1)	
- Missing	14 (19.4)	
Treatment		
- Yes	53/72 (73.6)	Three HW reported to result negative at a second H.pylori test (false negative at the HPOS screening); two HW were not prescribed treatment by the GP because asymptomatic. One HW among those prescribed with treatment by the GP explained that, the a first contact, the GP reported that it’s been too long since H.pylori diagnosis to undergo a treatment; at a second GP contact, based on a new positive test, the patient was recommended the treatment but preferred not to undergo it.
- No	5/72 (6.9)	
- Missing	14/72 (19.4)	
Type of treatment		
- 10 days sequential therapy	25/72 (34.7)	
- 10 days concomitant/	20/72 (27.8)	
- quadruple therapy	3/72 (4.2)	
- 14 days triple therapy	1/72 (1.4)	
- Modified triple therapy	2/72 (2.8)	
- Hybrid therapy	21/72 (29.2)	
- Missing		
Probiotics prescription		
- No	27/72 (37.5)	
- Yes	26/72 (36.1)	
- Missing	19/72 (26.4)	
Hp test repeated after treatment		
- No	13/72 (18.1)	The repetition of the H.pylori test for eradication confirmation was recommended by the research team at the time of results communication (by phone and by e-mail). Some participants did not repeat the test yet at the time of the follow-up, despite they retrieved information with the purpose to repeat it. At least 40 confirmatory tests were performed (type of tests: 19 SAT; 21 UBT). At the time of the follow-up, 37/40 HW received the result of the confirmatory test and reported it at the questionnaire. One HW asked to the research team to repeat the test after treatment because the GP was too busy.
- Yes	40/72 (55.6)	
- Missing	19/72 (26.4)	
Eradication		33/37 (89.1%) HW resulted negative after first-line treatment and 3/37 (8.1%) HWs were eradicated after second-line treatments. Out of 37 HW who reported the result of the confirmatory test, eradication rate was 97.3% with one HW not eradicated because of treatment interruption due to allergic reaction.
- No	26/72 (36.1)	
- Yes	36/72 (50.0)	
- Missing	10/72 (13.9)	
• **Eradication rate restricted to HW with result of a confirmatory Hp test:**		
- No	1/37 (2.7)	
- Yes	36/37 (97.3)	
Adverse effects		The same HW could have reported multiple symptoms One subject reported intense diarrhea and allergic reaction which required to interrupt the treatment. One reported extrasystole and tachycardia during clarithromycin + metronidazole, which disappeared when the treatment finished. 23 additional participants reported the following disturbs: eight epigastric pain/burn; seven weakness; three abdominal bloating; two stipsis; two increased heart rate; one smell-related discomfort.
- Diarrhea	12/72 (16.7)	
- Altered feces color	18/72 (25.0)	
- Altered taste	10/72 (13.9)	
- Headache/dizziness	11/72 (15.3)	
- Nausea	24/72 (33.3)	
- Other disturbs	23/72 (31.9)	
- Missing	19/72 (26.4)	
Duration of adverse effects		
- 1–3 days	10/72 (13.9)	
- 4–7 days	14/72 (19.4)	
- 7–14 days	16/72 (22.2)	
- Missing	32/72 (44.4)	
When adverse effects		
- During treatment	20/72 (27.8)	
- During and after treatment (up to 7 days after the end)	20/72 (27.8)	
- Only after the treatment	1/72 (1.4)	
- Missing	31/72 (43.1)	
Difficulty of the treatment		
- No	7/72 (9.7)	
- Small	13/72 (18.1)	
- Moderate	25/72 (34.7)	
- High	5/72 (6.9)	
- Missing	22/72 (30.6)	
Treatment compliance		Four HW referred to have forgotten one dose of the treatment; one HW referred to have forgotten two doses; one HW referred to have assumed two doses with a delay.
- Complete	43/72 (59.7)	
- Incomplete	6/72 (8.3)	
- Missing	23/72 (31.9)	
Attitude of the physician in terms of interest and availability		
- Scarce	2/72 (2.8)	
- Moderate	24/72 (33.3)	
- High	14/72 (19.4)	
- Missing	32/72 (44.4)	
The physician informed the patient about the importance of treatment compliance		
- No	4/72 (5.6)	
- Yes	46/72 (63.9)	
- Missing	10/72 (13.9)	
Clinical change after treatment		Of those who reported any symptom, 26.0% reported improvement, 7.4% reported changes but not as improvement.
- No	33/72 (45.8)	
- Yes, improvement	12/72 (16.7)	
- Yes, but not as improvement	3/72 (4.2)	
- Missing	24/72 (33.3)	
Mean study rate 1–10	8.9 (8.6–9.2)	Based on 48 observations. The lowest score was 5 (one participant); most common score was 9 (19/48, 39.6%) followed by 10 (16/48, 33.3%).
Usefulness of the Hp screening proposed		
- Low	4/72 (5.6)	
- Medium	7/72 (9.7)	
- High	40/72 (55.6)	
- Missing	21/72 (29.2)	
Would you suggest the HP screening?		
- No	9/72 (12.5)	
- Yes	40/72 (55.6)	
- Only in symptomatic	1/72 (1.4)	
- Missing	22/72 (30.6)	
Families’ involvement		28 HW showed interest; 13 HW screened the families, including adult siblings and/or husband or sister/brother; 16 HW did not suggest the screening to families, including four HW who reported the partner has been already tested in the past.
- No	20/72 (27.8)	
- Yes	28/72 (38.9)	
- Missing	24/72 (33.3)	
Feedback and suggestions from the HW	Full management under the occupational medicine service, including treatment and *H.pylori* test after treatment; avoid GP involvement; inform about the benefit of probiotics; improve communication between occupational medicine unit and gastroenterologist/laboratory; clarity in the information about how to repeat the test after the treatment; improve the setting of the intervention; implement the *H.pylori* screening as a novel service within the HW; implement other cancer screening at the workplace; add another clinical appointment in addition to the follow-up phone call.	

Notes: N, number; H.pylori, Helicobacter pylori; HW, hospital worker; GP, general practitioner

A total of 53/72 (73.6) *H.pylori*-positive HWs were prescribed an eradication treatment, while five were not (6.9%) either because they were negative at a second *H.pylori* test (*n* = 3) or were asymptomatic (*n* = 2) and therefore, according to the GP, did not need treatment.

One participant who was finally prescribed eradication therapy reported a two-stage interaction with their GP. The initial consultation resulted in a clinical delay, as the GP deferred treatment citing an excessive latency period since the primary diagnosis of infection. Eradication therapy was subsequently recommended during a secondary consultation following a new positive diagnostic test; however, the patient ultimately exhibited treatment hesitancy and declined the prescription.

Overall, 34.7% underwent the 10 days sequential therapy and 27.8% the 10 days concomitant/quadruple therapy; the remaining received either 14 days triple therapy, modified triple therapy or hybrid therapy. Furthermore, 26/72 HWs (36.1%) were prescribed probiotics next to eradication treatment.

At the time of follow-up, 40/72 HWs (55.6%) have repeated a confirmatory test after treatment. Overall, 36/72 *H.pylori*-positive HWs were successfully eradicated (50.0%). In particular, among 37 HWs with confirmatory test results after treatment, 33/37 (89.1%) were eradicated with first-line treatment while 3/37 (8.1%) turned negative after second-line therapy; eradication rate was 97.3%, with one out of 37 HWs having interrupted the treatment because of an allergic reaction.

Data on adverse effects of the treatment were available for 50/72 HWs. The most commonly reported conditions were nausea (31.9%) and altered feces color (25.0%), while 16.7% had diarrhea. Among the relevant conditions, one subject reported intense diarrhea and one reported an allergic reaction leading to treatment interruption; another manifested extrasystole and tachycardia during the treatment, yet none needed hospitalization. Most treated HWs referred complete compliance with the therapy (43/72, 59.7%). Most treated HWs (25/72, 34.7%) judged the therapy moderately difficult, while 6.9% reported high difficulty.

Factors related to the GP indicated a generally positive attitude toward the proposed screening, with a focus on recommending treatment adherence. However, a lack of sufficient information regarding potential adverse effects was reported.

When asked about their screening experience, HWs included in the follow-up rated it positively (mean score: 8.9/10) and considered it very useful (40/72, 55.6%). The majority stated they would recommend *H.pylori* screening (40/72, 55.6%). Finally, 28/72 HWs (38.9%) expressed interest in involving their family members in the screening process; among them, 13 had one or more family members screened (18.0% over the total HWs screened positive).

## Discussion

We implemented the first real-world *H.pylori* screening program among HWs in Italy, representing the first workplace-based initiative for GC prevention, achieving a nearly 75% participation rate. An additional group of HWs was enrolled as volunteers, showing strong interest toward the program. Higher participation was observed among women and nurses, while men and physicians were less likely to be successfully engaged. *H.pylori* prevalence was 16.6%, lower than expected. The three-month follow-up evidenced that most positive HWs referred to their GP, who was often favorable to the proposed screening. We recorded around 30% incomplete adherence to recommendations at follow-up, often due to procrastination of *H.pylori* management after diagnosis. Nevertheless, the eradication rate was 97.3% among those treated. A subgroup of *H.pylori*-positive HW underwent upper endoscopy, with one HW diagnosed with erosive gastritis as the most severe finding. The *H.pylori* screening was well received, with most HWs rating it highly useful and indicating they would recommend it.

To our knowledge, this is one of the few workplace-based *H.pylori* screenings implemented globally and the first in Italy. Zober et al. assessed *H.pylori* prevalence via serology among 6,143 chemical workers in Germany as a health promotion initiative within the OHS in 1995–1996 [24]. In particular, 4,488 were recruited during OHS and 1,655 on a voluntary basis. However, the participation rate is unclear, as the authors did not specify the total number of invited workers [24]. Madisch et al. conducted a *H.pylori* screening using a urea breath test among German factory workers who were referred to the OM center for dyspepsia [25]. Of 526 workers who contacted the center, 110 were ineligible, 149 refused and 267 were enrolled, corresponding to 64.2% of participation rate [25].

Our study corroborates the hypothesis that the workplace might be a suitable setting for the implementation of an *H.pylori* screening [4, 14, 15]. Many HWs valued the chance to undergo an additional health test during OHS, and multiple HWs interviewed at follow-up referred that they would recommend *H.pylori* screening during OHS as part of the standard practice. This favorable attitude reflects alignment with the concept of TWH [2–4].

*H.pylori* screening gains value from its unique nature of a one-time, lifelong preventive measure for GC if eradication is achieved, since the infection is mainly acquired in early childhood and reinfection is unlikely [8, 14].

The success of this intervention can be attributed to different strengths. Informative materials (e.g., poster, brochures, hospital webpage) likely encouraged participation and word-of-mouth dissemination. The high engagement suggests that HWs perceived the screening as a valuable opportunity for a free, voluntary health check-up rather than a corporate requirement. Participants were assured that their test results were confidential and independent from their fitness to work, ensuring privacy protection.

The hospital setting facilitated adherence in all phases [3, 14], and ensured the infrastructures for implementation [3, 14]. Proximity between the OM Unit and the laboratory promoted timely SAT transfer. Similarly, the multidisciplinary expertise of occupational physicians and gastroenterologists fostered trust and engagement.

The lower participation rate of men aligns with data showing less engagement in preventive programs compared to women [26–28]. In particular, female sex was associated with higher participation in a previous *H.pylori* screening among 40–64-year-old individuals from Latvia [28]. This supports findings that men underestimate health risks and show reluctance toward medical contact [29, 30]. Lower participation among physicians and researchers likely reflects time constraints and busy schedules. Adherence to the HPOS protocol was challenged by time-sensitive SAT collection. We adopted SAT rather than serological tests for its (i) higher sensitivity and specificity than serology [19, 20] and (ii) established cost-effectiveness for screening strategies [8]. Moreover, ethical considerations related to the post-COVID-19 pandemic prevented the use of the urea breath test [31].

Overall, participation rate was surprisingly high, especially when compared to that reported in other studies in Europe [28, 32]. This might be due to the workplace setting, the specific design of the protocol, and the targeted population. In fact, not only the hospital setting may have facilitated the access of HWs to the OM Unit where the screening was delivered, but HWs also represent a special group with higher health literacy and therefore more prone to engage in health initiatives. The higher dropout rates observed in physicians/researchers might be explained by time constraints and a scarce perception of their risk status. The multiple invitation strategies also represented important facilitators of the successful implementation of this intervention [33]. Preliminary results from the CPW study [34] confirm that *H.pylori* screening programs yield higher satisfaction and approval rates from occupational health providers when conducted in hospital settings (Mean = 4.29) compared to corporate environments (Mean = 3.92). Future CPW analyses will provide further data on participation in *H.pylori* screening across diverse working populations [15].

The *H.pylori* treatment in Italy is covered by the National Health System when prescribed by the GP, with small variations by Region in terms of ticket price [35]. The high referral rate to gastroenterologists over the GP is attributed to the study’s implementation within S. Orsola University Hospital, which has a strong historical focus on gastroenterology, alongside a protocol offering direct specialist access under request.

The observed *H.pylori* prevalence was lower than suggested in the literature but consistent with the declining trend of *H.pylori* infection which has been widely described [36]. Despite certain HWs categories, such as endoscopy staff, being at higher risk [22], no pattern was detected, likely due to the small subgroup size. An association with educational level was observed, as in previous studies [37, 38].

A relatively high proportion of HWs were unaware of *H.pylori* infection and related health risks. This confirms the persisting knowledge gap about this carcinogenic bacterium even among health professionals [9–12]. The finding also reinforces the educational value of workplace health promotion, empowering workers and communities for *H.pylori* control and GC prevention [4, 15].

Follow-up was incomplete for several HWs, partly due to missed contact and management delays. Overall, most of the followed-up positive HW referred to a physician, which was a fundamental step for *H.pylori* eradication. The GP referral was often unsatisfactory, showing procrastination from both patients and physicians. The treatment regimen used in this study was largely based on current guidelines [8]. Although some studies have shown that probiotics may have a certain auxiliary effect on the bactericidal activity of *H.* *pylori*, the prescription method is not yet consistent [8, 39]. During the treatment, although some patients experienced recurrent symptoms, only one patient discontinued treatment as a result. Our study describes real-world *H*. *pylori* management in Italy in 2020s, showing divergences from guidelines in terms of treatment prescription, eradication scheme, and confirmatory test after treatment [35]. While this aspect is a strength of the present study, the results point out the need for a standardized and more guidelines-adherent pathway of *H*. *pylori* management [35]. The implementation of a large-scale *H.pylori* screening may be supported by structured, informed management of the infection, to be adapted to the local context in order to account for possible antibiotic resistance patterns [4, 14, 33, 34].

Building upon these single-center findings, the ongoing multicenter European project CPW is currently evaluating the large-scale feasibility of integrating *H.* *pylori* screening programs within routine OHS across diverse European workplace environments [15]. CPW aims to provide comprehensive implementation and participation data across heterogeneous working populations, distinct national healthcare pathways, and varying infection management models [15, 34].

The HWs resulting positive were informed of the possible higher risk of *H.pylori* infection in their first-degree families based on evidence on early childhood transmission and were advised to discuss their testing with the GP for evaluation, especially if symptomatic. In fact, the follow-up interview revealed that *H.pylori* screening was extended to some family members, confirming a broader public health potential of the workplace-based intervention. While a protocol involving secondarily the family members of *H.pylori*-positive workers helps in the strategic identification of individuals at higher risk of infection and in reducing the reservoir of infection [4, 14, 40], it should not imply a certainty of *H.pylori* positivity in first-degree families or close contacts. Since *H.pylori* transmission occurs rarely in adulthood and under standard hygienic conditions [41], family involvement should be framed and offered as a preventive awareness measure rather than an assumption of household occurrence of infection [14].

Few HW reported a noticeable clinical improvement. A prolonged longitudinal observation (e.g., 12–24 months) would better capture changes in health status, and several years are needed to assess the GC prevention impact [42]. *H. pylori* screening may also reduce benign conditions such as gastritis, a common cause of sick leave and reduced productivity [43]. Thus, workplace-based *H. pylori* screening could yield occupational benefits [43]. This area remains largely unexplored [43]. In fact, workplace-based *H.pylori* screening might convey occupational benefits, such as reduced absenteeism for sick leave due to *H. pylori*-related disease and impaired work performance. This aspect is, to date, largely unexplored [43], requiring longitudinal studies.

A prolonged follow-up of the population participating to* H. pylori* screen-and-treat strategies would evidence changes in *H. pylori*-related diseases. In Italy, 58.8% of GC has been estimated to be attributable to *H.pylori* infection in 2040, corresponding to more than 4,400 deaths in men and more than 3,000 in women if the infection were eradicated [44]. The replication of the present study will allow collection of important data for cost-effectiveness analyses and for informing policy makers on the public impact of such prevention strategies on overall health and GC in particular [44].

This study has some limitations. The low *H.pylori* prevalence we observed resulted in low statistical power, limiting the identification of patterns of risk of *H.pylori* infection in the study population. Analyses were further impaired by the incomplete follow-up data. While the 3-month time point offers an early post-treatment picture, a longer observation would reveal delayed health effects. Moreover, while this environment fostered a highly sensitized population and streamlined care for *H.pylori* positives, it limits the protocol’s generalizability to non-hospital settings. However, the currently ongoing workplace-based *H.pylori* screening within the CPW project [15], which is an expanded adaptation of the HPOS study, supports the scalability of the present screening strategy across different settings and European populations. Finally, to minimize missing data, participants short on time were allowed to complete the questionnaire at home. While this maximized the response rate, it may have introduced a degree of response bias among this specific subgroup.

In conclusion, the HPOS study demonstrated the feasibility of integrating a workplace-based *H.pylori* screening within routine OHS in an Italian hospital, achieving high participation and strong acceptance. Although *H.pylori* prevalence was relatively low, most infected individuals consulted a physician following the diagnosis and underwent eradication therapy consistent with current guidelines. Several family members of *H.pylori*-positive HWs were also tested through local health system.

This study carries important implications for implementing workplace-based *H.pylori* screening strategies. Our approach could be reproduced in other occupational settings, as in the ongoing CPW project interventions [15]. Expanding such screening across occupational groups could help understand the determinants of participation and guide engagement strategies. Future studies should investigate the impact of workplace-based *H.pylori* screening on absenteeism, productivity and wellbeing.

## Supplementary Information

Below is the link to the electronic supplementary material.

Supplementary file1 (DOCX 53 KB)

Supplementary file2 (PDF 135 KB)

## Data Availability

The original de-identified data will be made available upon reasonable request.

## Abbreviations

CI: Confidence interval
CRF: Case report form
EU-OSHA: European agency for occupational safety and health at work
GC: Gastric cancer
GP: General practitioner
*H.pylor**i*: *Helicobacter pylori*
HPOS: *Helicobacter pylori* Screening project on healthcare workers
HWs: Hospital workers
OHS: Occupational health surveillance
OM: Occupational medicine
SAT: Stool antigen test
TWH: Total worker health
