# Supplementary material for: A real-world workplace-based screening for Helicobacter pylori infection: the HPOS study
Source: Cancer Causes Control. 2026 Jul 30;37(9):139. doi: 10.1007/s10552-026-02189-2 (PMC13424376; doi:10.1007/s10552-026-02189-2)
Supplement: Supplementary file 1 — Supplementary file1 (DOCX 53 KB) [file 10552_2026_2189_MOESM1_ESM.docx]

**Supplementary materials**

Supplementary table 1. STROBE checklist.

| Section | Item No. | Recommendation | Page |
| --- | --- | --- | --- |
| Title and abstract | 1 | (a) Indicate the study’s design with a commonly used term in the title or the abstract; (b) Provide in the abstract an informative and balanced summary of what was done and what was found | 1-3 |
| Introduction | 2 | Explain the scientific background and rationale for the investigation being reported | 7 |
| Introduction | 3 | State specific objectives, including any prespecified hypotheses | 7 |
| Methods | 4 | Present key elements of study design early in the paper | 7 |
| Methods | 5 | Describe the setting, locations, and relevant dates, including periods of recruitment, exposure, and data collection | 7,8 |
| Methods | 6 | Give the eligibility criteria, and the sources and methods of selection of participants | 7,8 |
| Methods | 7 | Clearly define all outcomes, exposures, predictors, potential confounders, and effect modifiers. Give diagnostic criteria, if applicable | 7,10 |
| Methods | 8 | For each variable of interest, give sources of data and details of methods of assessment (measurement). Describe comparability of assessment methods if there is more than one group | 10 |
| Methods | 9 | Describe any efforts to address potential sources of bias | NA |
| Methods | 10 | Explain how the study size was arrived at | 7 |
| Methods | 11 | Explain how quantitative variables were handled in the analyses. If applicable, describe which groupings were chosen and why | 10 |
| Methods | 12 | (a) Describe all statistical methods, including those used to control for confounding; (b) Describe any methods used to examine subgroups and interactions; (c) Explain how missing data were addressed; (d) Describe analytical methods taking account of sampling strategy; (e) Describe any sensitivity analyses | 10 |
| Results | 13 | (a) Report numbers of individuals at each stage of study; (b) Give reasons for non-participation at each stage; (c) Consider use of a flow diagram | 11-13, Tables 1-3 |
| Results | 14 | (a) Give characteristics of study participants (e.g., demographic, clinical, social) and information on exposures and potential confounders; (b) Indicate number of participants with missing data for each variable of interest | 11, Table 1 |
| Results | 15 | Report numbers of outcome events or summary measures | 11-13, Tables 1-3 |
| Results | 16 | (a) Give unadjusted estimates and, if applicable, confounder-adjusted estimates and their precision (e.g., 95% CI); (b) Report category boundaries when continuous variables were categorized; (c) If relevant, consider translating estimates of relative risk into absolute risk for a meaningful time period | NA |
| Results | 17 | Report other analyses done—e.g., analyses of subgroups and interactions, and sensitivity analyses | NA |
| Discussion | 18 | Summarise key results with reference to study objectives | 14 |
| Discussion | 19 | Discuss limitations of the study, taking into account sources of potential bias or imprecision. Discuss both direction and magnitude of any potential bias | 17 |
| Discussion | 20 | Give a cautious overall interpretation of results considering objectives, limitations, multiplicity of analyses, results from similar studies, and other relevant evidence | 18 |
| Discussion | 21 | Discuss the generalisability (external validity) of the study results | 17 |
| Other information | 22 | Give the source of funding and the role of the funders for the present study and, if applicable, for the original study on which the present article is based | 2 |

**Supplementary table 2. Knowledge and history of Helicobacter pylori by selected characteristics**

| **Characteristic** | **N (%)**  **494 (100%)*** |
| --- | --- |
| H.pylori knowledge   - No - Yes | 32 (6.5)  462 (93.5) |
| In your opinion, what is the level of knowledge of H.pylori among general population?   - Known to most - Very well known - Well known - Poorly known - Quite unknown - Missing | 5 (1.0)  11 (2.2)  169 (34.2)  188 (38.1)  100 (20.2)  21 (4.3) |
| Where did you get to know H.pylori?   - General practitioner - Studies/workplace - Family - Television - Internet - Books/journals - Missing | 33 (6.7)  358 (72.5)  41 (8.3)  9 (1.8)  6 (1.2)  20 (4.0)  27 (5.5) |
| First-degree family member H.pylori?   - No - I do not know - Yes - Missing | 282 (57.1)  117 (23.7)  85 (17.2)  10 (2.0) |
| First-degree family member GC?   - No - I do not know - Yes - Missing | 392 (79.40)  36 (7.3)  56 (11.3)  10 (2,02) |

Notes: N, number; Hp, Helicobacter pylori; GC, gastric cancer

*Numbers may not sum up because of missing data

**APPENDICES**

1. **Informative document: background of the HPOS study.**
2. **Information for study participants**
3. **Consent form**
4. **Baseline questionnaire**
5. **Follow-up questionnaire**

**A- Informative materials: background of the HPOS study.**

**Helicobacter pylori screening among hospital workers – Prospective observational study conducted on a population of Hospital Workers**

Dear Sir/Madam,

We illustrate you a new study entitled "Helicobacter pylori screening among hospital workers – Prospective observational study conducted on a population of Hospital Workers," promoted by University of Bologna.

The main objective of this study is to determine the prevalence of Helicobacter pylori (Hp) infection in hospital workers (HW), identifying individuals at higher risk of diseases related to the bacterium.

Additional objectives are to analyze the risk factors related to Hp infection in HW and to evaluate the feasibility of a screening protocol for Hp infection in a work environment, studying its impact on the prognosis and potential health benefits for the individual.

Helicobacter pylori infection is widespread globally, with prevalence rates reaching up to 50% in developing countries, especially in areas with poor hygiene conditions and inadequate medical treatment. Hp is a significant risk factor for chronic gastritis and gastric ulcers. Additionally, the International Agency for Research on Cancer (IARC) has classified Hp as a Group 1 carcinogen due to its association with gastric cancer.

Although most infected individuals remain asymptomatic, some may develop severe conditions such as gastric cancer. Given the potentially severe health outcomes and the possibility of effective antibiotic treatment, early detection and treatment of Hp infection are essential for prevention.

Study Design and Methods

The study will involve a sample of approximately 496 healthcare workers aged between 40 and 65 years, employed at the S. Orsola Hospital and referred to the Occupational Medicine Department for routine occupational health surveillance. The study will span two years as an observational prospective study.

The protocol involves testing for Hp infection through stool antigen test. Participants will receive a sample collection kit, and results will be communicated via email and/or phone. Participants who test positive will be informed of the therapeutic options available and referred to a medical specialist if needed.

Participation and Consent

Participation in the study is voluntary. Participants may withdraw their consent at any time without providing any justification, and this will not affect their future healthcare or employment.

Participants will be informed of their results, and in the case of a positive diagnosis, the research team will provide guidance on the next steps and therapeutic interventions.

Key Benefits of the Study

• Non-invasive testing: Diagnosis will be conducted using a non-invasive stool antigen test.

• Targeted Population: The study will focus on hospital workers aged 40-65 during their routine occupational health visits.

• Risk Reduction: Early detection and treatment can eliminate a risk factor for gastric cancer and ensure effective prevention.

For further information or questions regarding the study, participants may contact the research team during the entire duration of the study.

The participant’s general practitioner will evaluate the eradication therapy. In case of necessity, the research team may also refer the participant to the study’s medical specialists for further consultations or specialized gastroenterological evaluations regarding Hp infection. Additionally, participants who test positive will be encouraged to have family members screened, as they represent a high-risk population for Hp infection.

Workers who test positive will be contacted again three months after the infection is identified to assess their general health status and gather information on the therapeutic pathway undertaken.

Diagnosis of Hp infection allows to identify subjects at increased risk of gastric cancer, which develops over decades and results from the combination of several factors. Cancer, although rare, occurs in a minority of cases and is preceded by easily detectable precancerous lesions during an endoscopic examination. Invasive investigation might be requested based on the single case, such as warning symptoms (eg., unexplained weight loss or anemia); some laboratories recommend an esophagogastroduodenoscopy if the patient is over 45 years old.

A negative Hp test excludes a significant risk factor for gastric cancer over the individual's lifetime. The eradication of the infection and its follow-up reduce the risk of developing gastric cancer by healing chronic mucosal inflammation and, in most cases, reversing precancerous lesions.

The detection of Hp infection represents a starting point to improve the individual’s health and well-being and allows the early identification of a major risk factor of stomach disease. The worker can make informed decisions regarding their health status through specialist consultations.

**B-INFORMATION FOR STUDY PARTICIPANTS**

**Helicobacter pylori screening among hospital workers – Prospective observational study conducted on a population of Hospital Workers**

For further information, please contact Dr. Giulia Collatuzzo (giulia.collatuzzo3@ unibo.it) or Dr. Giulia Fiorini (giulia.fiorini@aosp.bo.it)

Dear Colleague,

Your collaboration is very important; therefore, we kindly ask you to read this information sheet carefully. We and our collaborators are available for any clarifications. Helicobacter pylori (Hp) is a bacterium associated with stomach diseases such as gastritis and ulcers. The infection is also linked to gastric cancer, of which it is the main risk factor. The study will assess the prevalence of Hp infection in a hospital environment and identify related factors. The hypothesis is that healthcare workers are, on one hand, at higher risk Hp infection, and on the other hand, have multiple protective factors such as hygienic habits and greater health awareness.

• USE OF THE STOOL COLLECTION KIT

Upon providing informed consent, you received a stool collection kit. This kit, similar to the fecal occult blood test kit, consisting of a cylindrical container with a screw cap equipped with a small scoop for stool collection. Use the kit as follows: after defecation, open the container by unscrewing the cap, taking care not to drop it and avoiding touching the scoop. Holding the cap, collect a stool sample using the scoop and place it into the container. Close the container by inserting the scoop with the collected stool sample and screw the cap back on. Wrap the securely closed container thoroughly with aluminum foil or plastic wrap.

It is essential that the collected sample is kept in the fridge until delivery and that delivery occurs within 2 days from the collection to ensure the validity of the test result. If the collection occur a few hours before sample delivery (e.g., the morning), it is sufficient to keep the sample in the refrigerator for a few minutes or a couple of hours.

• DELIVERY OF THE CONTAINER

The container should be delivered on Monday, Tuesday, Wednesday, or Thursday (not on Friday) between 8:00 AM and 2:00 PM at PAVILION 9, FIRST FLOOR. Therefore, avoid collecting the sample on Thursday and Friday, as it cannot be delivered before the following Monday, which would be 3–4 days after collection.

• TEST RESULT

The test result will be provided in approximately 14 days, depending on the workload of the analysis laboratory.

The test results will be the only communication of the outcome for healthcare workers who test negative for Hp. Workers who test positive for Hp will be contacted by the researchers by phone and will receive appropriate information regarding possible diagnostic-therapeutic pathways; a Hp positivity record will be sent following the phone call.

1. **Consent form**

**CONSENT FORM**

Protocol for screening for Helicobacter pylori screening among hospital workers – Prospective observational study conducted on a population of Hospital Workers

I, the undersigned,

born on ..................................................................................... in .......................................................................................

residing at ........................................................................................., .......................................

telephone ...................................................................................., email ..............................................................,

hereby declare:

• I have received thorough explanations regarding the request to participate in the study, particularly concerning its objectives and the procedures.

• I was informed of the opportunity to ask questions and that I received satisfactory answers.

• I have read and understood the information provided to me and I was given sufficient time to consider it.

• I understand that participation is voluntary and I may withdraw from the study at any time, without needing to provide an explanation and without it affecting my future medical care.

• I am aware that if I withdraw my consent, the data collected until the withdrawal will be retained and used in accordance with the research protocol.

• I am also aware of the possibility that if the test result is positive, the medical doctor will contact me with a letter regarding the result.

Consequently, based on these declarations:

• I voluntarily agree to participate in the study.

• I agree to be contacted in the future to provide follow-up information.

Name & Surname: ..............................................................................

Date .....................................................................................................

Signature: ......................................................................................

Name of the person collecting the consent: ............................................................

Date ....................................................................................................

Signature: ......................................................................................

**D- BASELINE QUESTIONNAIRE**

**GENERAL AND GASTROENTEROLOGICAL HEALTH STATUS ASSESSMENT QUESTIONNAIRE**

HPOS study: Prospective observational study conducted on a population of healthcare professionals.

The following questionnaire is aimed at collecting sociodemographic, behavioral and clinical information.

Please clearly indicate your answers, whether positive or negative, by entering a cross or another sign in the corresponding box. If you are uncertain about an answer, please leave it blank or indicate 'I don't know’.

When you find written "specify", write down your answer in the space marked by ellipsis.

Identification number (assigned by the researcher; also to be reported on the second page)

..................................................................

NAME AND SURNAME ......

ID CODE

Mobile phone number.........

DATE OF COMPILATION.........

TIME……

N identifier .........

GENERAL AND EMPLOYMENT DATA

A1 Date of birth ............ A2 Place of birth......

A3 Height .............................. A4 Weight ...............

A5 Current municipality of domicile .............. A6 from the year......

A7 Level of education:

1. Elementary/Middle School (or Lower) |____|

2. Technical/professional high school |____|

3. High School |____|

4. Graduation |____|

5. Doctorate/Master/ Postgraduate Qualifications |____|

A8 Employment qualification

6. Doctor/MFS. |____|

7. Nurse |____|

8. Technician |____|

9. Administrative |____|

10. OSS |____|

11. Other (please specify) ............

A9 Department (please specify) ............

A10 Specific tasks...

A11 Start of work in the University Hospital S.Orsola-Malpighi (year) ..............................

A12 Previous occupations and their period (specify type of work and years during which it was carried out)

..................................................................................................................................................................

........................................................................................................................................

A13 In your work, do you regularly perform or assist investigative/interventional and invasive procedures that may cause contact with the oral cavity or anogenital organs? (examples: intubation, patient hygiene, bladder catheterization, removal of bladder catheter, gynecological examination...)

YES |____|

NO |_____|

A14 Do you ever work/have you worked for at least 1 month in a digestive or respiratory endoscopy department?

YES |____|

NO |_____|

A15 You perform your activities mainly in:

CLINICAL WARD |_____|

OUTPATIENT CLINIC |_____|

LABORATORY |_____|

SERVICES (e.g. canteen, bar, transport) |_____|

OFFICE |_____|

A16 Marital status

MARRIED OR COHABITING |____|

SEPARATE |____|

SINGLE |____|

WIDOWER |____|

A17 Do you have children?

NO |___

YES |_____| How many? ..................... What sex? ......................

A18 What are your habits with respect to cigarette smoking?

NEVER SMOKED |__|

EX SMOKER |__| Specify for how many years ........................ How many years ago did he stop................

SMOKER |__| Please specify how many years........................ How many cigarettes a day........................

A19 Do you do sports?

NONE OR ALMOST NONE |__|

LIGHT (e.g. walks) |__|

MODERATE (e.g. sport 1-2 times a week) |__|

INTENSE (e.g. sport 3 or more times a week) |__|

KNOWLEDGE ABOUT HELICOBACTER PYLORI

B1 Have you ever heard or read about helicobacter pylori?

NO |____|  go to the next section (Diet)

YES |____|  Continue with this section.

B2 If you know about Helicobacter pylori, in what context did you first learn about it?

DURING STUDIES OR IN A WORK ENVIRONMENT |_____|

FROM THE ATTENDING PHYSICIAN |____|

FAMILY |_____|

ON THE INTERNET |_____|

ON TELEVISION |______|

ON BOOKS/MAGAZINES/NEWSPAPERS |______|

B3 In your opinion, how well known is Helicobacter pylori infection in the general population?

RATHER UNKNOWN |____|

VERY LITTLE KNOWN |____|

FAIRLY KNOWN |____|

WELL KNOWN |____|

IT IS KNOWN TO MOST PEOPLE |____|

B4 Have you ever tested for Helicobacter pylori infection?

NO |____|

YES |____|

B5 Have you ever been treated for Helicobacter pylori infection?

NO |____|YES |____| How many days did it last?

7 DAYS |____|

14 DAYS |____|

OTHER (please specify)...............

DIET

Food Unit Number of units per week (if not at all, enter 0)

E1 1 cup Milk |__| __|

E2 1 portion Yoghurt |__| __|

E3 1 portion Pasta/rice |__| __|

E6 1 serving Red meat |__| __|

E7 1 serving Chicken |__| __|

E7 1 serving Fish (not canned) |__| __|

E8 1 serving Canned fish/in oil |__| __|

E9 1 serving Sausages |__| __|

E10 1 plate Vegetable |__| __|

E11 1 medium fruit, 2 small fruits Fruit |__| __|

E12 1 serving Biscuits/sweets/dessert |__ |__|

E13 1 unit Coffee |__| __|

E14 1 glass Wine |__ |__|

E15 1 can (33 cl) Beer |__| __|

Does E16 Generally add salt to food?

NO |__|

YES |__|

E17 Are there any foods you usually avoid? Specify......................................................…

PHARMACOLOGICAL HISTORY

Q1 Do you usually use probiotics (generally prescribed for seasonal cycles, e.g. Prolactis, Enterolactis, Reuterin, Lactoflorene, Prolife, VSL3...)?

No |__|

Yes |__|

Q2 Do you use antithyroid drugs (e.g. Levothyroxine)?

No |__|

Yes |__|

Q3 Do you use iron supplements?

No |__|

Yes |__|

Q4 Do you use corticosteroids or other immunosuppressants in chronic?

No |__|

Yes |__|

Q5 in the last year, how many times have you used antibiotics (eg. For respiratory or urinary infections)?

Never |__|

1-2 |__|

3 or more |__|

Q6 in the previous 5 years, how many times do you estimate you have used antibiotics in a year?

Never |__|

Less than 1 time per year |__|

1-2 times a year |__|

3 or more times a year |__|

GENERAL PATHOLOGICAL HISTORY

P1 Do you suffer from thyroid disease?

NO |__|YES |__|

P2 Do you suffer from iron deficiency anemia?

NO |__|YES |__|

P3 Do you suffer from dermatitis?

NO |__|

YES |__| specify.................................................

P4 Do you have allergies?

NO |__|

YES |__| specify....................................................

P5 Do you suffer from headache/migraine?

NO |__|YES |__|

P6 Do you suffer from inflammatory bowel disease (Chron's disease, ulcerative colitis)?

NO |__|YES |__|

P7 Have you been diagnosed with celiac disease?

NO |__|

YES |__|

P8 Have you been diagnosed with diabetes?

NO |__|

YES |__|

P9 Have you been diagnosed with hypertension?

NO |__|

YES |__|

P10 Do you suffer from hematological diseases?

NO |__|

YES |__| specify........................................................................................

P10 Do you suffer from other chronic diseases? Specify..........................................................

PATHOLOGICAL HISTORY SPECIFIC TO THE GASTROINTESTINAL TRACT

G1 Do you Suffer/have you ever suffered from gastro-oesophageal reflux?

NO |__|

YES |__|

G2 Do you ever suffer/have you suffered from gastritis?

NO |__|

YES |__|

G3 Have you ever been diagnosed with peptic ulcer?

NO |__|

YES |__|

G4 Have you ever had a gastroscopy?

NO |__|

YES |__| Have you underwent gastroscopy in the last 5 years?

NO |__|

YES |__|

What diagnostic outcome? Specify.................................

G5 In the last 3 months, have you had abdominal pain?

NO |__|YES |__| How often?

DAILY |__|

ABOUT 1 TIME PER WEEK |__|

ABOUT 1 TIME PER MONTH |__|

How intense was the abdominal pain?

MILD |__|

MODERATE |__|

INTENSE |__|

Marks in the image in which quadrants he had pain

[image]

G6 How often you have had each of the following symptoms in the past 3 months

1. Epigastric pain/discomfort:

never |__|

<1 time per month |__|

1 time per month |__|

1 time per week |__|

1 time per week-1 time per day |__|

1 or more times a day |__|

2. Retrosternal burning

never |__|

<1 time per month |__|

1 time per month |__|

1 time per week |__|

1 time per week-1 time per day |__|

1 or more times a day |__|

3. Regurgitation

never |__|

<1 time per month |__|

1 time per month |__|

1 time per week |__|

1 time per week-1 time per day |__|

1 or more times a day |__|

4. Nausea

never |__|

<1 time per month |__|

1 time per month |__|

1 time per week |__|

1 time per week-1 time per day |__|

1 or more times a day |__|

5. Which of these symptoms has been most disturbing in the last 2 months?

None |__|

Epigastric pain/discomfort |__|

Retrosternal burning |__|

Regurgitation |__|

Nausea |__|

G7. Do you have at least one first-degree relative with a history of gastric cancer?

NO |__|

I DON'T KNOW |__|

YES |__|Specify how many and the type of kinship......

G8. Do you have at least one first-degree relative who has been diagnosed with Helicobacter pylori infection?

NO |__|

I DON'T KNOW |__|

YES |__| Specify how many and the type of kinship .......

**E- Follow-up questionnaire**

**FOLLOW-UP QUESTIONNAIRE**

The following questionnaire includes follow-up questions regarding the fecal test for Helicobacter pylori (Hp) antigens, which you took as part of the HPOS study – a screening study on Helicobacter pylori among healthcare workers.

The questions aim to investigate the diagnostic and therapeutic path you may have undertaken after the detection of Hp infection, as well as your opinions on this process and your participation in the study.

Full name:

Date of birth:

Did you contact your general practitioner after receiving the Hp test result?

• ☐ NO

• ☐ YES

• ☐ Researchers from the team contacted them for me

• ☐ I consulted another doctor (e.g., gastroenterologist)

Were you prescribed antibiotic therapy for Hp eradication?

• ☐ NO

• ☐ OTHER NON-ANTIBIOTIC MEDICATIONS (please specify): ___________________________

• ☐ YES – Which one?

o 14-day triple therapy

o 10-day sequential therapy

o 10-day concomitant/quadruple therapy with bismuth

o

Did you experience any discomfort or symptoms related to the therapy?

• ☐ NO / VERY MILD

• ☐ YES, MILD TO MODERATE

• ☐ YES, MODERATE TO SEVERE

• ☐ YES, SEVERE ENOUGH TO DISCONTINUE THE TREATMENT

If yes, what kind of symptoms did you experience?

• ☐ Diarrhea

• ☐ Discoloration of stool

• ☐ Headache / dizziness

• ☐ Altered taste

• ☐ Nausea / loss of appetite / heaviness

• ☐ Severe allergy / severe diarrhea / other complications

• ☐ Other (please specify): ____________________________________________

When did the symptoms occur?

• ☐ DURING THE THERAPY

• ☐ DURING AND AFTER THE THERAPY (up to 7 days later)

• ☐ ONLY AFTER THE THERAPY

How long did the symptoms last?

• ☐ 1–3 days

• ☐ 4–7 days

• ☐ 7–14 days

Were you informed by your doctor about possible side effects?

• ☐ NO

• ☐ YES

Did your doctor explain the importance of strictly following the prescribed therapy?

• ☐ NO

• ☐ YES

Were you prescribed any other medications along with the therapy?

• ☐ NO

• ☐ YES, TO TAKE PROBIOTICS (please specify): ___________________________

• ☐ YES, OTHER (please specify): ______________________________________

Did you ever forget to take a pill (e.g., missed doses or skipped a day)?

• ☐ NO

• ☐ YES – How many? ___________________________

Did you find the therapy burdensome?

• ☐ NO

• ☐ A LITTLE

• ☐ QUITE

• ☐ VERY MUCH

Did you undergo a second test for Helicobacter pylori to confirm eradication?

• ☐ NO – Did your doctor advise against repeating it (or did you refuse)? ______________________

• ☐ YES – When? (date if possible): ___________________________

Which test? ___________________________________

Did you notice any changes in your gastrointestinal health after the therapy?

• ☐ NO

• ☐ YES, IMPROVEMENT

• ☐ YES, BUT NOT AN IMPROVEMENT

What was your general practitioner's attitude toward your process?

• ☐ NOT VERY INTERESTED OR AVAILABLE

• ☐ QUITE INTERESTED AND AVAILABLE

• ☐ VERY INTERESTED AND AVAILABLE

How useful do you think this path has been for you?

• ☐ NOT MUCH

• ☐ QUITE

• ☐ VERY MUCH

Overall, how would you rate the experience you had through this study, from 1 (poor) to 10 (excellent)?

Do you have any notes or suggestions about the proposed process?

Would you recommend this pathway to your acquaintances?

Some of your first-degree relatives (adult children, siblings up to 65 years old, parents up to 65 years old – note: not your partner due to lack of transmission risk) can be included in this study and take the same diagnostic test (fecal test for Hp) through occupational health services. Would you be interested in offering this to any of them?

Who? ___________________________________________________________________________

**APPENDIX F**

Operational details for replication of the intervention.

| **Study phase** | **Time burden** | **Staff roles** |
| --- | --- | --- |
| Recruitment | About 3 hours each morning, Monday-Friday; about 10 minutes per participant, including 2-3 minutes for standardized oral study presentation and 2-3 minutes for following consent form collection and delivery of informative materials | One or two trained researchers (eg., referent researchers or trained resident physicians in Occupational Medicine) in each recruitment day.  Involvement of occupational physician (eg., presentation of the study, referral to the researchers), occupational nurses (eg., acceptance of volunteers, referral to the researchers, collection of HW names or contact information when needed, referral to the room for stool sample preservation in the fridge). |
| Volunteer involvment | Immediate management when the HW referred in person to the OM Unit during the recruitment.  Some minutes when the HW contacted the researchers via e-mail or by calling the OM Unit (management usually within days from the contact). | Two referent researchers available for e-mail contact. Occupational nurses present at acceptance of OM Unit. |
| Baseline questionnaire administration | About 20 minutes, in the waiting room or the dedicated study room; the HW could also bring home the questionnaire (adaptation requested based on time constraints of the HW). | One researcher remained available for potential clarifications during the questionnaire filling; the questionnaire filling of a participant usually took place while waiting for the planned occupational health visit, and during the recruitment of other potential HW. |
| SAT reception and transportation to the lab | SAT could be returned every morning; for organizational reasons, HW were asked not to return the SAT on Fridays, to avoid missing the lab analysis by 48 hours from the collection.  SAT were transferred with a cooler bag from the fridge of the OM Unit to that of the lab (100 meters) on Tuesdays and Thursdays. | Occupational nurses and researchers managed the return of SAT and the correct placement in the fridge.  One researcher or trained resident physician transferred the SAT to the lab every Tuesdays and Thursdays when closing the recruitment session. The lab professional was notified about the sample delivery and their number. |
| SAT analysis | Analytical turnaround time of approximately 20 minutes. Two days a week dedicated to sample analyses in the lab (eg. 1 hour each). | One dedicated laboratory professional, notified when samples were available for analyses.  The lab professional also reported the test result in the dedicated excel file when ready. |
| Result communication | Negative results communicated via e-mail.  Positive results communicated by phone call and then by e-mail, with written report (about 10-20 minutes each positive HW). | E-mail sent on a regular basis when several results were collected.  Positive results were prioritized and communicated ideally within 3 days from analysis result.  One researcher or trained occupational physician called by phone the Hp positive HW and communicated the results and implications, providing recommendations on Hp management and referring the HW to the GP. A standardized explanation of about 5 minutes was offered, but the researcher addressed possible HW’s questions and doubts. The referent researchers remained available for Hp positive HW following the positive test and before the follow-up. |
| Follow-up questionnaire | 10-15 minutes, possibly varying based on case-specific situation or needs | One dedicated trained researcher for phone calls and data collection. A gastroenterologist from the reseach team remained available for further exams and counseling upon request. |
| Reminders to participants missing to return SAT and missing follow-up | About 1 hour per week, during specific project phases (eg., at 3—6 months from recruitment; at 3 months from SAT positivity) | One researcher or trained resident physician for Periodical reminders by phone call (up to three times per participant) |
