# Supplementary material for: A real-world workplace-based screening for Helicobacter pylori infection: the HPOS study
Source: Cancer Causes Control. 2026 Jul 30;37(9):139. doi: 10.1007/s10552-026-02189-2 (PMC13424376; doi:10.1007/s10552-026-02189-2)
Supplement: Supplementary file 2 — Supplementary file2 (PDF 135 KB) [file 10552_2026_2189_MOESM2_ESM.pdf]

HPOS STUDY

Giulia Collatuzzo, Giulia Fiorini, Matteo Pavoni, Dino Vaira, Paolo Boffetta

APPENDICES

- **A- Informative document: background of the HPOS study.**
- **B- Consent form**
- **C- Baseline questionnaire**
- **D- Follow-up questionnaire**

A- Informative materials: background of the HPOS study.

Helicobacter pylori screening among hospital workers – Prospective observational study conducted on a population of Hospital Workers

Dear Sir/Madam,
We illustrate you a new study entitled "Helicobacter pylori screening among hospital workers – Prospective observational study conducted on a population of Hospital Workers," promoted by University of Bologna.
The main objective of this study is to determine the prevalence of Helicobacter pylori (Hp) infection in hospital workers (HW), identifying individuals at higher risk of diseases related to the bacterium.
Additional objectives are to analyze the risk factors related to Hp infection in HW and to evaluate the feasibility of a screening protocol for Hp infection in a work environment, studying its impact on the prognosis and potential health benefits for the individual.

Helicobacter pylori infection is widespread globally, with prevalence rates reaching up to 50% in developing countries, especially in areas with poor hygiene conditions and inadequate medical treatment. Hp is a significant risk factor for chronic gastritis and gastric ulcers. Additionally, the International Agency for Research on Cancer (IARC) has classified Hp as a Group 1 carcinogen due to its association with gastric cancer.
Although most infected individuals remain asymptomatic, some may develop severe conditions such as gastric cancer. Given the potentially severe health outcomes and the possibility of effective antibiotic treatment, early detection and treatment of Hp infection are essential for prevention.
Study Design and Methods
The study will involve a sample of approximately 496 healthcare workers aged between 40 and 65 years, employed at the S. Orsola Hospital and referred to the Occupational Medicine Department for routine occupational health surveillance. The study will span two years as an observational prospective study.
The protocol involves testing for Hp infection through stool antigen test. Participants will receive a sample collection kit, and results will be communicated via email and/or phone. Participants who test positive will be informed of the therapeutic options available and referred to a medical specialist if needed.

<u>Participation and Consent</u>
Participation in the study is voluntary. Participants may withdraw their consent at any time without providing any justification, and this will not affect their future healthcare or employment.
Participants will be informed of their results, and in the case of a positive diagnosis, the research team will provide guidance on the next steps and therapeutic interventions.

<u>Key Benefits of the Study</u>
- Non-invasive testing: Diagnosis will be conducted using a non-invasive stool antigen test.
- Targeted Population: The study will focus on hospital workers aged 40-65 during their routine occupational health visits.
- Risk Reduction: Early detection and treatment can eliminate a risk factor for gastric cancer and ensure effective prevention.

For further information or questions regarding the study, participants may contact the research team during the entire duration of the study.

The participant’s general practitioner will evaluate the eradication therapy. In case of necessity, the research team may also refer the participant to the study’s medical specialists for further consultations or specialized gastroenterological evaluations regarding Hp infection. Additionally, participants who test positive will be encouraged to have family members screened, as they represent a high-risk population for Hp infection.

Workers who test positive will be contacted again three months after the infection is identified to assess their general health status and gather information on the therapeutic pathway undertaken. Diagnosis of Hp infection allows to identify subjects at increased risk of gastric cancer, which develops over decades and results from the combination of several factors. Cancer, although rare, occurs in a minority of cases and is preceded by easily detectable precancerous lesions during an endoscopic examination. Invasive investigation might be requested based on the single case, such as warning symptoms (eg., unexplained weight loss or anemia); some laboratories recommend an esophagogastroduodenoscopy if the patient is over 45 years old.

A negative Hp test excludes a significant risk factor for gastric cancer over the individual's lifetime. The eradication of the infection and its follow-up reduce the risk of developing gastric cancer by healing chronic mucosal inflammation and, in most cases, reversing precancerous lesions.
The detection of Hp infection represents a starting point to improve the individual’s health and well-being and allows the early identification of a major risk factor of stomach disease. The worker can make informed decisions regarding their health status through specialist consultations.

B- **Consent form**

CONSENT FORM

Protocol for screening for Helicobacter pylori screening among hospital workers – Prospective observational study conducted on a population of Hospital Workers
I, the undersigned,
born on .... in
....
residing at ...., ...
telephone ...., email
....,
hereby declare:

- I have received thorough explanations regarding the request to participate in the study, particularly concerning its objectives and the procedures.
- I was informed of the opportunity to ask questions and that I received satisfactory answers.
- I have read and understood the information provided to me and I was given sufficient time to consider it.
- I understand that participation is voluntary and I may withdraw from the study at any time, without needing to provide an explanation and without it affecting my future medical care.
- I am aware that if I withdraw my consent, the data collected until the withdrawal will be retained and used in accordance with the research protocol.
- I am also aware of the possibility that if the test result is positive, the medical doctor will contact me with a letter regarding the result.

Consequently, based on these declarations:

- I voluntarily agree to participate in the study.
- I agree to be contacted in the future to provide follow-up information.

Name & Surname: ....
Date ....
Signature: ....

Name of the person collecting the consent: ....
Date ....
Signature: ....

C- BASELINE QUESTIONNAIRE

GENERAL AND GASTROENTEROLOGICAL HEALTH STATUS ASSESSMENT QUESTIONNAIRE

HPOS study: Prospective observational study conducted on a population of healthcare professionals.

The following questionnaire is aimed at collecting sociodemographic, behavioral and clinical information.
Please clearly indicate your answers, whether positive or negative, by entering a cross or another sign in the corresponding box. If you are uncertain about an answer, please leave it blank or indicate 'I don't know'.
When you find written "specify", write down your answer in the space marked by ellipsis.

Identification number (assigned by the researcher; also to be reported on the second page)

....

NAME AND SURNAME ......

ID CODE

Mobile phone number.........

DATE OF COMPILATION.........

TIME……

N identifier ....

GENERAL AND EMPLOYMENT DATA

A1 Date of birth .... A2 Place of birth......
A3 Height .... A4 Weight ....

A5 Current municipality of domicile .... A6 from the year......

A7 Level of education:
1. Elementary/Middle School (or Lower) |____|
2. Technical/professional high school |____|
3. High School |____|
4. Graduation |____|
5. Doctorate/Master/ Postgraduate Qualifications |____|

A8 Employment qualification
6. Doctor/MFS. |____|
7. Nurse |____|
8. Technician |____|
9. Administrative |____|
10. OSS |____|
11. Other (please specify) ....

A9 Department (please specify) ....

A10 Specific tasks...

A11 Start of work in the University Hospital S.Orsola-Malpighi (year) ....

A12 Previous occupations and their period (specify type of work and years during which it was carried out)

....
..

....

A13 In your work, do you regularly perform or assist investigative/interventional and invasive procedures that may cause contact with the oral cavity or anogenital organs? (examples: intubation, patient hygiene, bladder catheterization, removal of bladder catheter, gynecological examination...)
YES |____|
NO |_____|

A14 Do you ever work/have you worked for at least 1 month in a digestive or respiratory endoscopy department?
YES |____|
NO |_____|

A15 You perform your activities mainly in:
CLINICAL WARD |_____|
OUTPATIENT CLINIC |_____|
LABORATORY |_____|
SERVICES (e.g. canteen, bar, transport) |_____|
OFFICE |_____|

A16 Marital status
MARRIED OR COHABITING |____|
SEPARATE |____|
SINGLE |____|
WIDOWER |____|

A17 Do you have children?
NO |___
YES |_____| How many? .... What sex? ....

A18 What are your habits with respect to cigarette smoking?
NEVER SMOKED |__|

EX SMOKER |__| Specify for how many years .... How many years ago did he stop................

SMOKER |__| Please specify how many years........................ How many cigarettes a day........................

A19 Do you do sports?
NONE OR ALMOST NONE |__|
LIGHT (e.g. walks) |__|
MODERATE (e.g. sport 1-2 times a week) |__|
INTENSE (e.g. sport 3 or more times a week) |__|

KNOWLEDGE ABOUT HELICOBACTER PYLORI
B1 Have you ever heard or read about helicobacter pylori?
NO |____| → go to the next section (Diet)
YES |____| → Continue with this section.

B2 If you know about Helicobacter pylori, in what context did you first learn about it?
DURING STUDIES OR IN A WORK ENVIRONMENT |_____|
FROM THE ATTENDING PHYSICIAN |____|
FAMILY |_____|
ON THE INTERNET |_____|
ON TELEVISION |______|
ON BOOKS/MAGAZINES/NEWSPAPERS |______|

B3 In your opinion, how well known is Helicobacter pylori infection in the general population?
RATHER UNKNOWN |____|
VERY LITTLE KNOWN |____|
FAIRLY KNOWN |____|
WELL KNOWN |____|

IT IS KNOWN TO MOST PEOPLE |____|

B4 Have you ever tested for Helicobacter pylori infection?
NO |____|
YES |____|

B5 Have you ever been treated for Helicobacter pylori infection?
NO |____|YES |____| How many days did it last?
7 DAYS |____|
14 DAYS |____|
OTHER (please specify)...............

DIET
Food Unit Number of units per week (if not at all, enter 0)

E1	1 cup	Milk	|__|	__|
E2	1 portion	Yoghurt	|__|	__|
E3	1 portion	Pasta/rice	|__|	__|
E6	1 serving	Red meat	|__|	__|
E7	1 serving	Chicken	|__|	__|
E7	1 serving	Fish (not canned)	|__|	__|
E8	1 serving	Canned fish/in oil	|__|	__|
E9	1 serving	Sausages	|__|	__|
E10	1 plate	Vegetable	|__|	__|
E11	1 medium fruit, 2 small fruits	Fruit	|__|	__|
E12	1 serving	Biscuits/sweets/dessert	|__	|__|
E13	1 unit	Coffee	|__|	__|
E14	1 glass	Wine	|__	|__|
E15	1 can (33 cl)	Beer	|__|	__|

Does E16 Generally add salt to food?
NO |__|
YES |__|

E17 Are there any foods you usually avoid? Specify...

PHARMACOLOGICAL HISTORY
Q1 Do you usually use probiotics (generally prescribed for seasonal cycles, e.g. Prolactis, Enterolactis, Reuterin, Lactoflorene, Prolife, VSL3...)?
No |__|
Yes |__|

Q2 Do you use antithyroid drugs (e.g. Levothyroxine)?
No |__|
Yes |__|

Q3 Do you use iron supplements?

No |__|
Yes |__|

Q4 Do you use corticosteroids or other immunosuppressants in chronic?
No |__|
Yes |__|

D5 in the last year, how many times have you used antibiotics (eg. For respiratory or urinary infections)?
Never |__|
1-2 |__|
3 or more |__|

Q6 in the previous 5 years, how many times do you estimate you have used antibiotics in a year?
Never |__|
Less than 1 time per year |__|
1-2 times a year |__|
3 or more times a year |__|

GENERAL PATHOLOGICAL HISTORY
P1 Do you suffer from thyroid disease?
NO |__|YES |__|

P2 Do you suffer from iron deficiency anemia?
NO |__|YES |__|

P3 Do you suffer from dermatitis?
NO |__|
YES |__| specify..

P4 Do you have allergies?
NO |__|
YES |__| specify...

P5 Do you suffer from headache/migraine?
NO |__|YES |__|

P6 Do you suffer from inflammatory bowel disease (Chron's disease, ulcerative colitis)?
NO |__|YES |__|

P7 Have you been diagnosed with celiac disease?
NO |__|
YES |__|

P8 Have you been diagnosed with diabetes?
NO |__|
YES |__|

P9 Have you been diagnosed with hypertension?
NO |__|

YES |__|

P10 Do you suffer from hematological diseases?
NO |__|
YES |__| specify...

P10 Do you suffer from other chronic diseases? Specify...

PATHOLOGICAL HISTORY SPECIFIC TO THE GASTROINTESTINAL TRACT

G1 Do you Suffer/have you ever suffered from gastro-oesophageal reflux?
NO |__|
YES |__|

G2 Do you ever suffer/have you suffered from gastritis?
NO |__|
YES |__|

G3 Have you ever been diagnosed with peptic ulcer?
NO |__|
YES |__|

G4 Have you ever had a gastroscopy?
NO |__|
YES |__| Have you underwent gastroscopy in the last 5 years?
NO |__|
YES |__|
What diagnostic outcome? Specify................................

G5 In the last 3 months, have you had abdominal pain?
NO |__|YES |__| How often?
DAILY |__|
ABOUT 1 TIME PER WEEK |__|
ABOUT 1 TIME PER MONTH |__|

How intense was the abdominal pain?
MILD |__|
MODERATE |__|
INTENSE |__|

Marks in the image in which quadrants he had pain

[image]

G6 How often you have had each of the following symptoms in the past 3 months
1. Epigastric pain/discomfort:
never |__|
<1 time per month |__|
1 time per month |__|

1 time per week |__|
1 time per week-1 time per day |__|
1 or more times a day |__|

2. Retrosternal burning
never |__|
<1 time per month |__|
1 time per month |__|
1 time per week |__|
1 time per week-1 time per day |__|
1 or more times a day |__|

3. Regurgitation
never |__|
<1 time per month |__|
1 time per month |__|
1 time per week |__|
1 time per week-1 time per day |__|
1 or more times a day |__|

4. Nausea
never |__|
<1 time per month |__|
1 time per month |__|
1 time per week |__|
1 time per week-1 time per day |__|
1 or more times a day |__|

5. Which of these symptoms has been most disturbing in the last 2 months?
None |__|
Epigastric pain/discomfort |__|
Retrosternal burning |__|
Regurgitation |__|
Nausea |__|

G7. Do you have at least one first-degree relative with a history of gastric cancer?
NO |__|
I DON'T KNOW |__|
YES |__|Specify how many and the type of kinship......

G8. Do you have at least one first-degree relative who has been diagnosed with Helicobacter pylori infection?
NO |__|
I DON'T KNOW |__|
YES |__| Specify how many and the type of kinship ....

D- Follow-up questionnaire

FOLLOW-UP QUESTIONNAIRE

The following questionnaire includes follow-up questions regarding the fecal test for Helicobacter pylori (Hp) antigens, which you took as part of the HPOS study – a screening study on Helicobacter pylori among healthcare workers.
The questions aim to investigate the diagnostic and therapeutic path you may have undertaken after the detection of Hp infection, as well as your opinions on this process and your participation in the study.

Full name:

Date of birth:

Did you contact your general practitioner after receiving the Hp test result?
- ☐ NO
- ☐ YES
- ☐ Researchers from the team contacted them for me
- ☐ I consulted another doctor (e.g., gastroenterologist)

Were you prescribed antibiotic therapy for Hp eradication?
- ☐ NO
- ☐ OTHER NON-ANTIBIOTIC MEDICATIONS (please specify):

____
- ☐ YES – Which one?
  - o 14-day triple therapy
  - o 10-day sequential therapy
  - o 10-day concomitant/quadruple therapy with bismuth
  - o

Did you experience any discomfort or symptoms related to the therapy?
- ☐ NO / VERY MILD
- ☐ YES, MILD TO MODERATE
- ☐ YES, MODERATE TO SEVERE
- ☐ YES, SEVERE ENOUGH TO DISCONTINUE THE TREATMENT

If yes, what kind of symptoms did you experience?
- ☐ Diarrhea
- ☐ Discoloration of stool
- ☐ Headache / dizziness
- ☐ Altered taste
- ☐ Nausea / loss of appetite / heaviness
- ☐ Severe allergy / severe diarrhea / other complications
- ☐ Other (please specify): ____

When did the symptoms occur?
- ☐ DURING THE THERAPY

- ☐ DURING AND AFTER THE THERAPY (up to 7 days later)
- ☐ ONLY AFTER THE THERAPY

How long did the symptoms last?
- ☐ 1–3 days
- ☐ 4–7 days
- ☐ 7–14 days

Were you informed by your doctor about possible side effects?
- ☐ NO
- ☐ YES

Did your doctor explain the importance of strictly following the prescribed therapy?
- ☐ NO
- ☐ YES

Were you prescribed any other medications along with the therapy?
- ☐ NO
- ☐ YES, TO TAKE PROBIOTICS (please specify): ___________________________
- ☐ YES, OTHER (please specify): ______________________________________

Did you ever forget to take a pill (e.g., missed doses or skipped a day)?
- ☐ NO
- ☐ YES – How many? ___________________________

Did you find the therapy burdensome?
- ☐ NO
- ☐ A LITTLE
- ☐ QUITE
- ☐ VERY MUCH

Did you undergo a second test for Helicobacter pylori to confirm eradication?
- ☐ NO – Did your doctor advise against repeating it (or did you refuse)?

____
- ☐ YES – When? (date if possible): ___________________________

Which test? ___________________________________

Did you notice any changes in your gastrointestinal health after the therapy?
- ☐ NO
- ☐ YES, IMPROVEMENT
- ☐ YES, BUT NOT AN IMPROVEMENT

What was your general practitioner's attitude toward your process?
- ☐ NOT VERY INTERESTED OR AVAILABLE
- ☐ QUITE INTERESTED AND AVAILABLE
- ☐ VERY INTERESTED AND AVAILABLE

How useful do you think this path has been for you?

- ☐ NOT MUCH
- ☐ QUITE
- ☐ VERY MUCH

Overall, how would you rate the experience you had through this study, from 1 (poor) to 10 (excellent)?

Do you have any notes or suggestions about the proposed process?

Would you recommend this pathway to your acquaintances?

Some of your first-degree relatives (adult children, siblings up to 65 years old, parents up to 65 years old – note: not your partner due to lack of transmission risk) can be included in this study and take the same diagnostic test (fecal test for Hp) through occupational health services. Would you be interested in offering this to any of them?
Who? ____
